# The Urine Proteome Profile Is Different in Neuromyelitis Optica Compared to Multiple Sclerosis: A Clinical Proteome Study

**DOI:** 10.1371/journal.pone.0139659

**Published:** 2015-10-13

**Authors:** Helle H. Nielsen, Hans C. Beck, Lars P. Kristensen, Mark Burton, Tunde Csepany, Magdolna Simo, Peter Dioszeghy, Tobias Sejbaek, Manuela Grebing, Niels H. H. Heegaard, Zsolt Illes

**Affiliations:** 1 Department of Neurology, Odense University Hospital, Odense, Denmark; 2 Institute for Clinical Research, University of Southern Denmark, Odense, Denmark; 3 Center for Clinical Proteomics, Odense University Hospital, Odense, Denmark; 4 Department for Clinical Genetics Odense University Hospital, Odense, Denmark; 5 Department of Neurology, University of Debrecen, Faculty of Medicine, Debrecen, Hungary; 6 Department of Neurology, Semmelweis University, Budapest, Hungary; 7 Department of Neurology, Josa Andras Teaching Hospital, Nyiregyhaza, Hungary; 8 Institute for Molecular Medicine, University of Southern Denmark, Odense, Denmark; 9 Department of Autoimmunology & Biomarkers, Statens Serum Institut, Copenhagen, Denmark; 10 Clinical Biochemistry, Institute of Clinical Research, University of Southern Denmark, Odense, Denmark; Medical University Vienna, Center for Brain Research, AUSTRIA

## Abstract

**Objectives:**

Inflammatory demyelinating diseases of the CNS comprise a broad spectrum of diseases like neuromyelitis optica (NMO), NMO spectrum disorders (NMO-SD) and multiple sclerosis (MS). Despite clear classification criteria, differentiation can be difficult. We hypothesized that the urine proteome may differentiate NMO from MS.

**Methods:**

The proteins in urine samples from anti-aquaporin 4 (AQP4) seropositive NMO/NMO-SD patients (n = 32), patients with MS (n = 46) and healthy subjects (HS, n = 31) were examined by quantitative liquid chromatography-tandem mass spectrometry (LC-MS/MS) after trypsin digestion and iTRAQ labelling. Immunoglobulins (Ig) in the urine were validated by nephelometry in an independent cohort (n = 9–10 pr. groups).

**Results:**

The analysis identified a total of 1112 different proteins of which 333 were shared by all 109 subjects. Cluster analysis revealed differences in the urine proteome of NMO/NMO-SD compared to HS and MS. Principal component analysis also suggested that the NMO/NMO-SD proteome profile was useful for classification. Multivariate regression analysis revealed a 3-protein profile for the NMO/NMO-SD versus HS discrimination, a 6-protein profile for NMO/NMO-SD versus MS discrimination and an 11-protein profile for MS versus HS discrimination. All protein panels yielded highly significant ROC curves (AUC in all cases >0.85, p≤0.0002). Nephelometry confirmed the presence of increased Ig-light chains in the urine of patients with NMO/NMO-SD.

**Conclusion:**

The urine proteome profile of patients with NMO/NMO-SD is different from MS and HS. This may reflect differences in the pathogenesis of NMO/NMO-SD versus MS and suggests that urine may be a potential source of biomarkers differentiating NMO/NMO-SD from MS.

## Introduction

Inflammatory demyelinating diseases of the CNS include putative autoimmune diseases like multiple sclerosis (MS), neuromyelitis optica (NMO), and NMO spectrum diseases (NMO-SD) e.g. relapsing and/or bilateral inflammatory optic neuritis (RION/BON), and longitudinally extensive transverse myelitis (LETM). Despite recognition of pathogenic antibodies against the water channel aquaporin 4 (AQP4) in the majority of patients with NMO/NMO-SD[1–3], diagnosis of especially seronegative cases can be challenging and underlines the need for additional biomarkers[4, 5]. Accurate diagnosis is nevertheless vital since misdiagnosis can lead to incorrect medication and deterioration[6].

Mass spectrometry has made it possible to uncover distinct molecular components in both serum and CSF of patients with NMO[7, 8] and MS[9]. Proteomic pattern analysis can globally and quantitatively characterize the protein population and may effectively reveal distinct and complex pathogenesis of seemingly closely related diseases, such as MS and NMO/NMO-SD. Previous non-quantitative studies have isolated biomarkers from CSF and serum[7, 8], while other body fluids remain to be been explored.

Urine has a relatively stable protein composition and may be obtained in large quantities non-invasively. It is considered as an attractive source of biomarkers[10] and the human urine proteome have been characterized by several techniques[11, 12]. Clinically applicable urine biomarkers have been identified even for diseases of the CNS[13].

In this study, we used high accuracy, high resolution quantitative mass spectrometry to characterize the urine proteome of healthy subjects (HS) and patients with seropositive NMO/NMO-SD and MS and investigated if the different pathophysiology of NMO may be reflected in the urine proteome profile.

## Materials and Methods

### Standard Protocol Approvals, Registrations, and Patient Consents

The study was approved by both the Hungarian National Ethics Committee (38.93.316-12464/KK4/2010, 42341-2/2013/EKU) as well as the Danish Ethics Committee of Region of Southern Denmark (S–20120066). Written consent was obtained from all participants prior to entering the study.

### Study Population

Using the NMO and MS databases of Odense University Hospital, Denmark and Pecs University, Hungary, we collected urine samples from 57 patients with AQP4-seropositive NMO/NMO-SD, 74 patients with relapsing-remitting MS (RR-MS) and 45 HS (table 1, **cohort 1**). For validation of Ig light chains in urine, an independent cohort of samples was collected (n = 9–10 pr group) (table 1, **cohort 2**). NMO/NMO-SD was diagnosed according to the Wingerchuk 2006[14] and the AQP4-seropositive NMO-SD criteria of EFNS[3], and their antibody status verified by a cell based assay (Euroimmune, Germany).

**Table 1:** Demographic data of cohorts.

**Cohort 1**			
	HS	AQP4-NMO/NMO-SD	MS
	n = 31	n = 32	n = 46
**Disease subtype**			
NMO	0	31	0
ON	0	0	0
LETM	0	1	0
RRMS	0	0	46
**Sex**			
Female	17	28	25
Male	14	4	21
**Mean age (range)**	40.2 (26–60)	44.5 (26–70)	42.2 (20–62)
**Treatment**			
Azathioprine	0	31	0
Natalizumab	0	0	24
Fingolimod	0	0	1
Interferon-beta	0	0	16
Glatiramer acetate	0	0	3
Dimethyl-fumerate	0	0	0
None of the above	31	1	3
**Cohort 2**			
	HS	AQP4-NMO/NMO-SD	MS
	n = 10	n = 9	n = 10
**Disease subtype**			
Definite NMO	0	9	0
ON	0	0	0
LETM	0	0	0
RRMS	0	0	10
**Sex**			
Female	5	9	7
Male	5	0	3
**Mean age (range)**	33.8 (26–53)	46.4(23–57)	38.2 (23–49)
**Treatment**			
Azathioprine	0	9	0
Natalizumab	0	0	0
Fingolimod	0	0	0
Interferon-beta	0	0	0
Glatiramer acetate	0	0	0
Dimethyl-fumerate	0	0	4
None of the above	10	0	6

NMO/NMO-SD, neuromyelitis optica/neuromyelitis optica spectrum disorder; MS, multiple sclerosis; HS, healthy subjects; ON, optic neuritis; LETM, longitudinally extensive transverse myelitis; RRMS, relapsing remitting multiple sclerosis.

All MS cases fulfilled the McDonald’s 2010 criteria[15]. HS did not suffer from autoimmune or neurological disorders (table 1). Neither MS nor NMO/NMO-SD patients had experienced a relapse within 30 days of the sample collection.

### Sample Preparation and Mass Spectrometry

**Fig 1:**
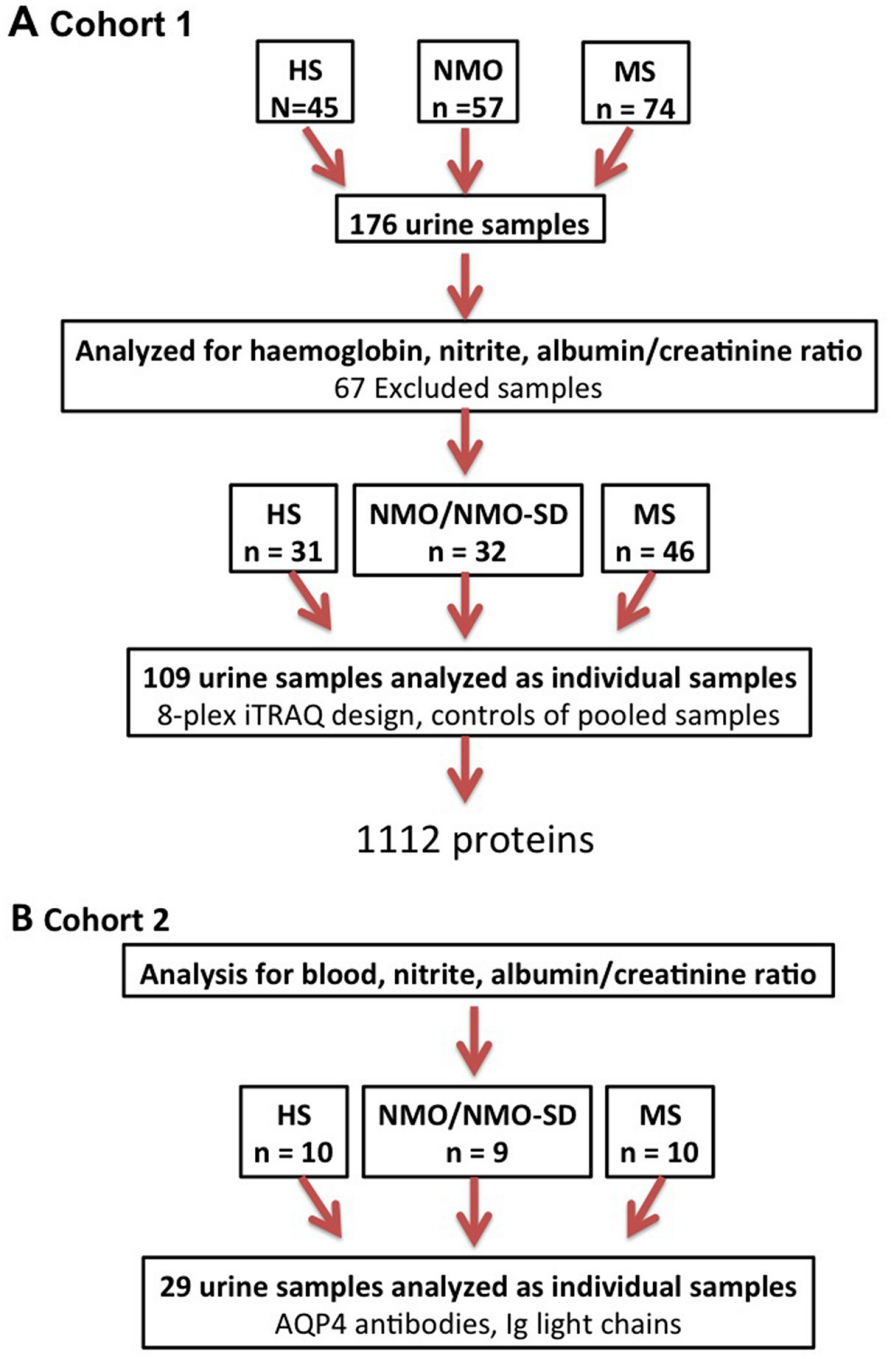
Outline of the study: enrolment and sample processing. Workflow and sample processing are shown. (A) Cohort 1: After initial screening and analysis of 176 urine samples, 67 samples were excluded due to presence of blood, nitrite, high albumin/creatinine ratio or low protein content. The remaining 109 samples were analysed as individual samples by quantitative LC-MS/MS proteomics compared to control groups of pooled samples of AQP4-seropositive NMO/NMO-SD, MS and HS samples, respectively, to yield 1112 proteins. (B) Cohort 2: For validation of increased Ig and AQP4 antibody excretion, samples from an independent cohort of patients with AQP4-seropositive NMO/NMO-SD (n = 9–10 pr group) were subjected to nephelometry and examination of anti-AQP4 by a cell-based assay. *NMO/NMO-SD*, *neuromyelitis optica/neuromyelitis optica spectrum disorder; MS*, *multiple sclerosis; HS*, *healthy subjects*.

Spot urine was collected and centrifuged within 2 hours before stored at -80°C until use. Samples containing blood, nitrite (Multistix 7, Siemens Healthcare), low protein content (<0.01 mg/ml), or displaying albumin/creatinine ratios >10 were excluded (Fig 1A). The sample cohort then consisted of 31 HS, 32 NMO/NMO-SD, and 46 MS samples.

Supernatants were filtered through 10 kDa cut-off spin-filters (Amicon). The retentates were washed in 500μl 5 mM triethylammonium bicarbonate buffer, re-suspended, reduced, alkylated and trypsinated[16]. Ten μg peptide aliquots were collected from each sample and labelled with isobaric tags (iTRAQ 8-plex): Mass tag 113 was assigned to 10μg of a HS pool; mass tag 114: 10μg of a NMO/NMO-SD pool; mass tag 115: 10μg of a MS pool; mass tags 116, 117, 118, 119, and 121: 10μg of randomly chosen HS, NMO/NMO-SD, and MS samples. The labelled samples were pooled into 24 8-plex sets, dried, re-dissolved in 0.1% trifluoroacetic acid, purified (WATERS, 5mg/well) and eluted[16] before separated into 11 fractions by hydrophilic interaction chromatography and analysed as previously described[16].

### Proteome Data Processing and Protein Quantification

A combined MASCOT-SEQUEST search was performed as described[16]. Tandem mass spectra were searched against the Swissprot database restricted to humans. Proteins were inferred on the basis of at least two unique peptides identified with high confidence. False-discovery rates (FDR) were obtained using Percolator selecting identification with a q-value ≤0.01. iTRAQ quantification was performed using Proteome Discoverer with reporter ion area integration within a 20ppm window. Ratios were normalized against the median peptide ratio. Ratios of diseased individuals (reporter ions 116–119 and reporter ion 121) versus the pool of HS (reporter ion 113) were used for statistical analysis. The ratios of HS (113) vs. NMO/NMO-SD pool (114) and MS pool (115) were used as a measure of the technical variability (≤8.1%).

#### Detection of immunoglobulins

Anti-AQP4 antibodies were detected using indirect immunofluorescence (Euroimmune, Germany) with crude samples or diluted 1:10, 1:20, and 1:40. For the detection of light chains, kappa (Ig-κ) and lambda (Ig-λ), samples from cohort 2 were analyzed by rate nephelometry (Siemens BN ProSpec instrument; detection limit: 7.11mg/L (Ig-κ) and 3.9mg/L (Ig-λ)), Ten NMO/NMO-SD samples from cohort 1 with high content of light chains served as positive controls (Fig 1B).

### Data Handling and Statistics

Relative intensities based on iTRAQ peptide counts in each sample group were tested for significant inter-group differences using unpaired two-sided t-tests in Microsoft Excel. The number of proteins found in more than 1 sample in each of the compared groups were: 1094 (NMO/NMO-SD vs. HS), 1075 (NMO/NMO-SD vs. MS), and 1029 (MS vs. HS), respectively. These numbers were used for the FDR adjustment. Data were z-transformed and hierarchically clustered using proteins present in >80% of the samples with p<0.05 and in subsequent analysis with FDR (q-values) <0.05[17, 18]. Principal components analysis (PCA) was performed as previously described[19].

For risk probability calculation and optimal discrimination of NMO/NMO-SD vs. HS, NMO/NMO-SD vs. MS, and MS vs. HS samples, respectively, logistic regression was applied. Input data were filtered according to different stringency criteria. Thus, modeling used expression values for: (1) proteins present in all samples (333 proteins); (2) proteins present in >80% of the samples in each group (520 proteins), and (3) proteins present in >2 samples in each group (1021 proteins). Missing values were assigned the value 0 for multivariate modeling. The feature selection and risk probability calculation were conducted as follows: 1) Calculating the univariate logistic regression based significance differentiating the 3 groups. 2) Ranking the proteins according to their univariate logistic regression significance. Modeling was limited to proteins with p≤0.05. 3) Developing logistic regression models differentiating either group[19]. Model performance was assessed by the area-under-curve (AUC) of the associated Receiver Operating Characteristic (ROC) curve and checked for invalidity as previously described[19].

## Results

### Protein Characteristics and Principal Component Analysis

**Fig 2:**
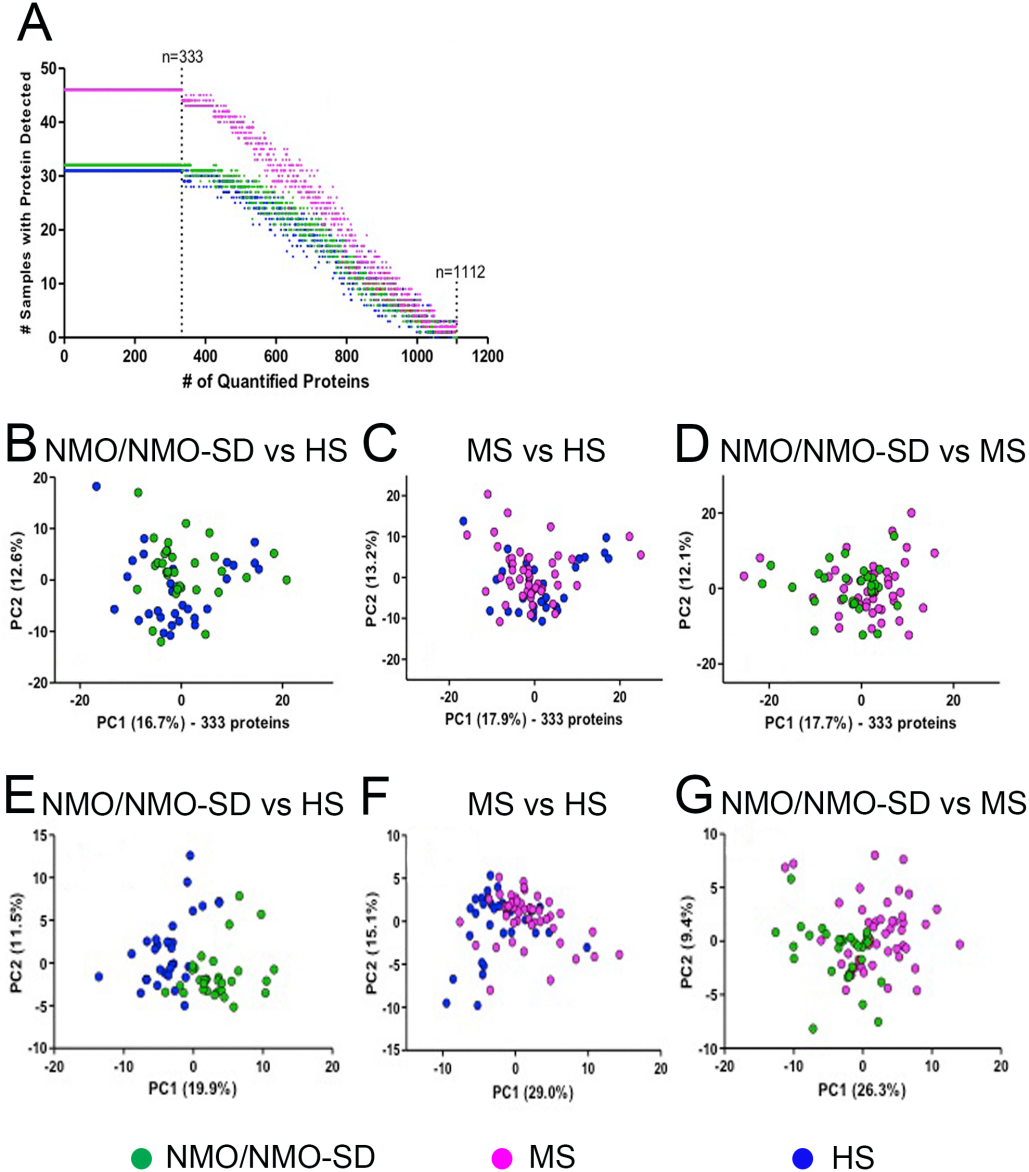
Cluster analysis of detected proteins in the urine comparing patients with NMO/NMO-SD, MS and healthy subjects. (A) Out of the 1112 proteins detected in the urine, 333 proteins were found in all samples. (B) PCA of all 333 proteins differentiated NMO/NMO-SD from HS samples. (C) MS samples could not be differentiated from HS by PCA. (D) NMO/NMO-SD samples could not be differentiated from MS samples by PCA. (E) PCA of proteins, which were differentially expressed (*p*<0.05) compared to HS and present in at least 80% of the samples enabled differentiation of the NMO/NMO-SD samples from HS. (F) PCA of proteins, which were differentially expressed (*p*<0.05) compared to HS and present in at least 80% of the samples enabled separation of the MS samples from HS. (G) PCA of proteins, which were differentially expressed (*p*<0.05) compared to HS and present in at least 80% of the samples enabled separation of the NMO/NMO-SD samples from MS. *PCA*, *principal component analysis; NMO/NMO-SD*, *neuromyelitis optica/neuromyelitis optica spectrum disorder; MS*, *multiple sclerosis; HS*, *healthy subjects*.

A total of 1112 proteins were identified by ≥2 peptides (Fig 1, S1 Table). Of these, 333 proteins were detected in all 109 samples (Fig 2A). By PCA of all 333 shared proteins, the two first principal components did not account for more than 30–33% of the variation. Except for the PCA plot of the NMO/NMO-SD and HS cases, no separation according to groups was apparent (Fig 2B–2D). However, when we included only proteins significantly expressed (*p*<0.05) compared to HS and present in >80% of the samples, the data contained information enabling the separation of the NMO/NMO-SD group from both HS and MS (Fig 2E and 2F, respectively), although less clear when comparing MS to HS (Fig 2G).

### FDR Adjustment Identifies Proteins Significant for NMO/NMO-SD and MS Discrimination

**Fig 3:**
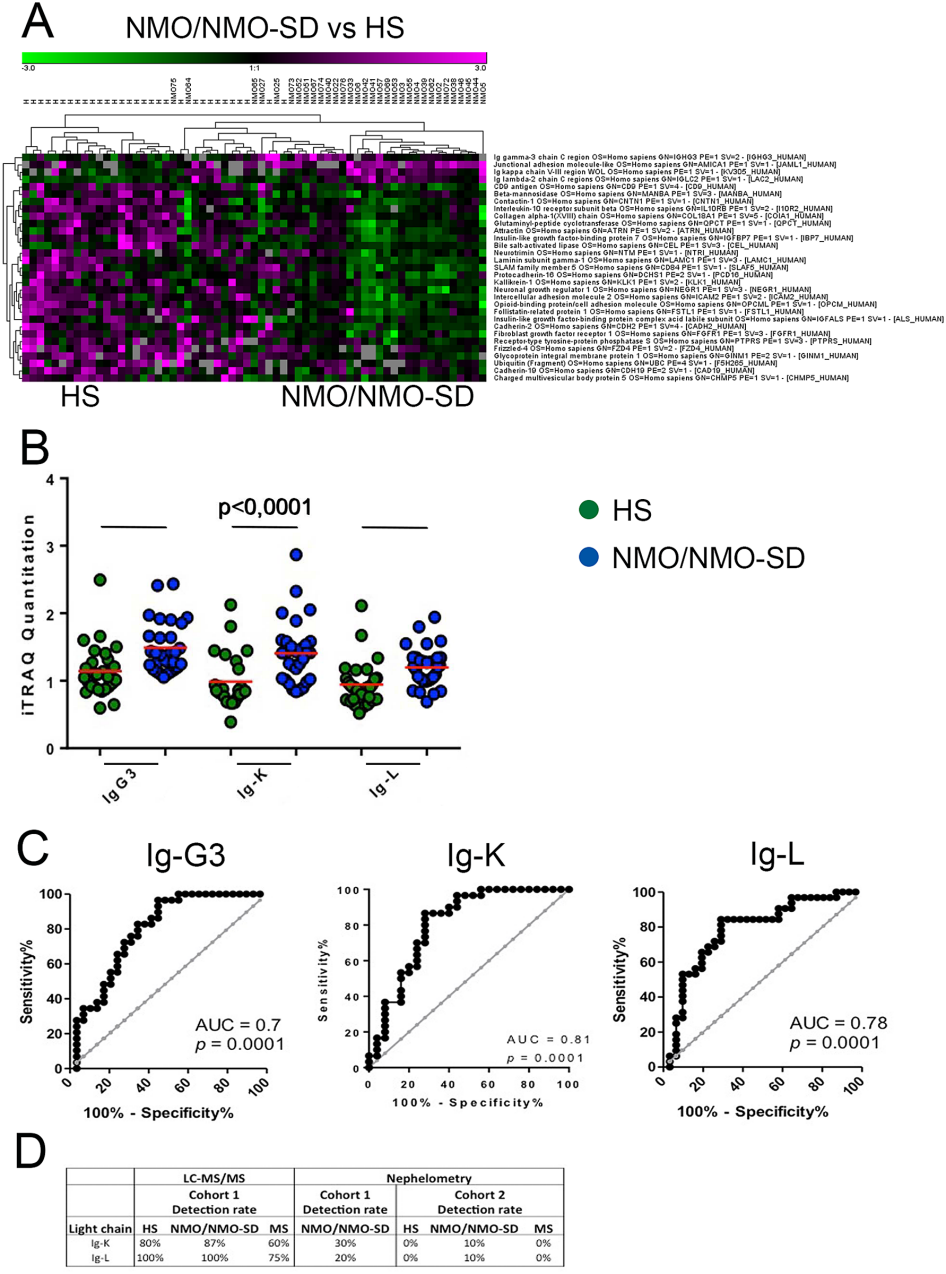
False Discovery Rate Adjustment identifies proteins significant for NMO/NMO-SD and HS discrimination in the urine. (A) Heat maps comparing NMO/NMO-SD and HS samples by false discovery rate adjustment with *q*-values less than 0.05 are shown. The analysis identified 31 proteins that discriminated NMO/NMO-SD from HS. (B) Only 3 fragments of Igs appeared to be upregulated compared to HS, the rest of the proteins were downregulated. (C) ROC curves for the Ig chains Ig-G3, Ig-K and Ig-L are shown. (D) Detection of Ig light chains by LC-MS/MS was 100% in Cohort 1, while only 20–30% by nephelometry. In cohort 2, nephelometry detected Ig light chains only in 10% of NMO/NMO-SD samples. *Magenta*, *upregulated compared to HS; Green*, *downregulated compared to HS; NMO/NMO-SD*, *neuromyelitis optica/neuromyelitis optica spectrum disorder; MS*, *multiple sclerosis; HS*, *healthy subjects; Ig-G3*, *immunoglobulin gamma–3 chain; Ig-K; immunoglobulin kappa chain; Ig-L*, *immunoglobulin lambda chain*.

We then compared NMO and MS samples using FDR adjustment to only allow entries with an FDR (q-value) <0.05 (Fig 3). In this way, we determined several proteins that discriminated NMO/NMO-SD from HS. The identified proteins are involved in biological functions such as leukocyte trafficking, demyelination and neuroinflammation (S2 Table). Three protein components of Igs (the gamma–3 (Ig-G3), Ig-κ and Ig-λ) were significantly upregulated in the NMO/NMO-SD samples compared to the HS group (Fig 3A and 3B), while others were downregulated. ROC curve analysis revealed AUC of 0.8 (p = 0.0001) in the NMO/NMO-SD vs HS comparison for all the three Igs (Fig 3C). Using an independent set of 10 samples from each of the three groups, we were unable to detect anti-AQP4 antibodies in urine by indirect immunofluorescence using M23 transfected cells. Although the light chains were detected in 20–30% of the NMO/NMO-SD samples from cohort 1 by nephelometry, only one NMO/NMO-SD sample (10%) contained Ig-κ and Ig-λ above the nephelometric detection threshold in cohort 2 (Fig 3D).

**Fig 4:**
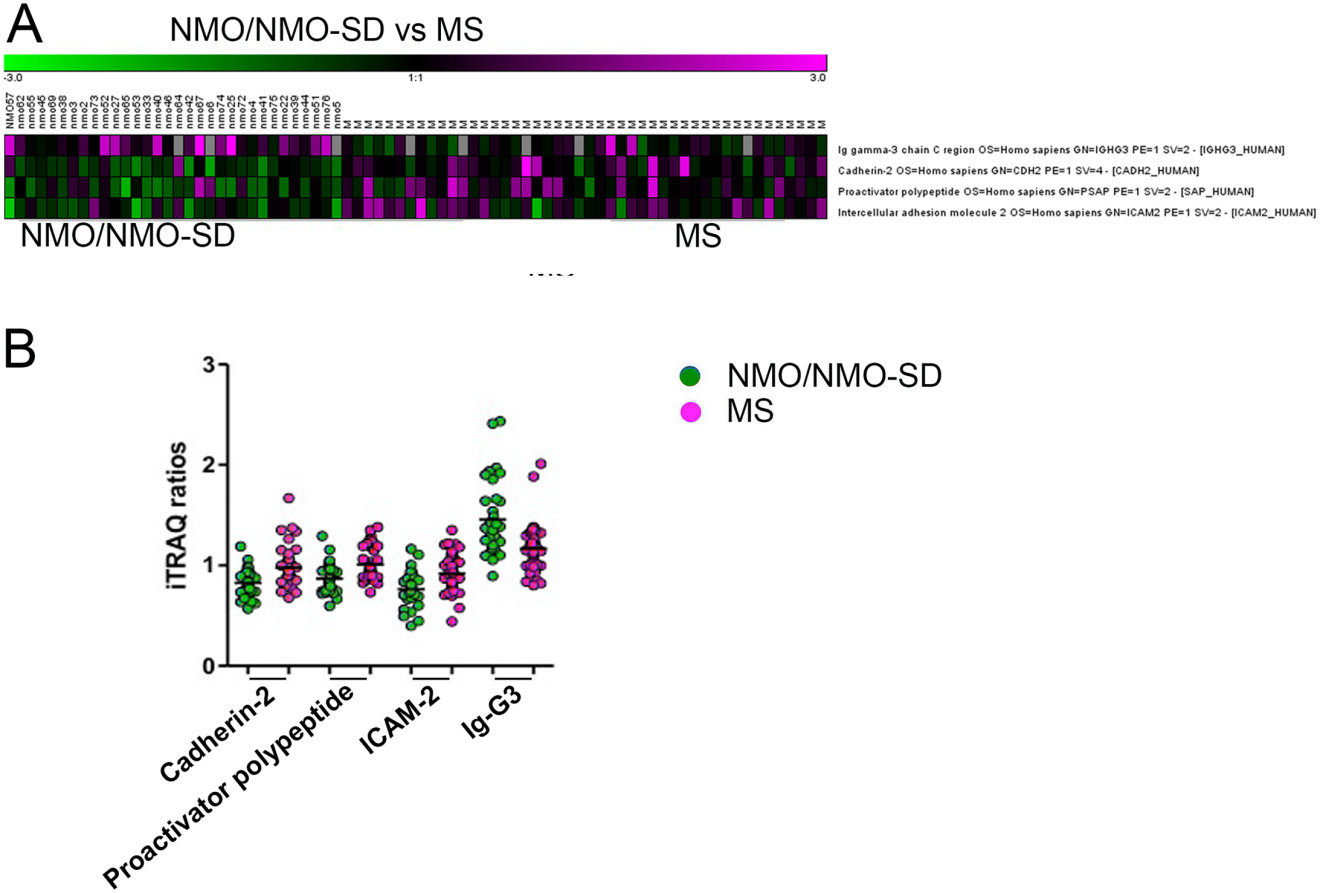
False Discovery Rate Adjustment identifies 4 proteins significant for NMO/NMO-SD and MS discrimination in the urine. A) Heat maps comparing NMO/NMO-SD and MS samples by false discovery rate adjustment with *q*-values less than 0.05 are shown. The analysis identified 4 proteins that discriminated NMO/NMO-SD from MS. (B) Only the protein Ig-G3 chain were found to be upregulated in NMO/NMO-SD compared to MS. *Magenta*, *upregulated compared to HS; Green*, *downregulated compared to HS; NMO/NMO-SD*, *neuromyelitis optica/neuromyelitis optica spectrum disorder; MS*, *multiple sclerosis; HS*, *healthy subjects; Ig-G3*, *immunoglobulin 3 chain; ICAM–2*, *Intercellular adhesion molecule*.

When adjusting for multiple comparisons at FDR <0.05, we identified proteins involved in leukocyte trafficking and myelin degeneration (S2 Table), which discriminated NMO/NMO-SD from MS (Fig 4A). IgG3 was significantly upregulated in NMO/NMO-SD compared to MS (Fig 4B) while the rest were all downregulated. Analysis of MS vs. HS revealed one discriminating protein (CD9), which was downregulated in MS compared to HS (S2 Table).

### Development of Diagnostic Classifiers

To optimize protein-based discrimination between NMO/NMO-SD, MS and HS from urine, we next applied logistic regression to check for protein combinations as potential diagnostic classifiers.

**Fig 5:**
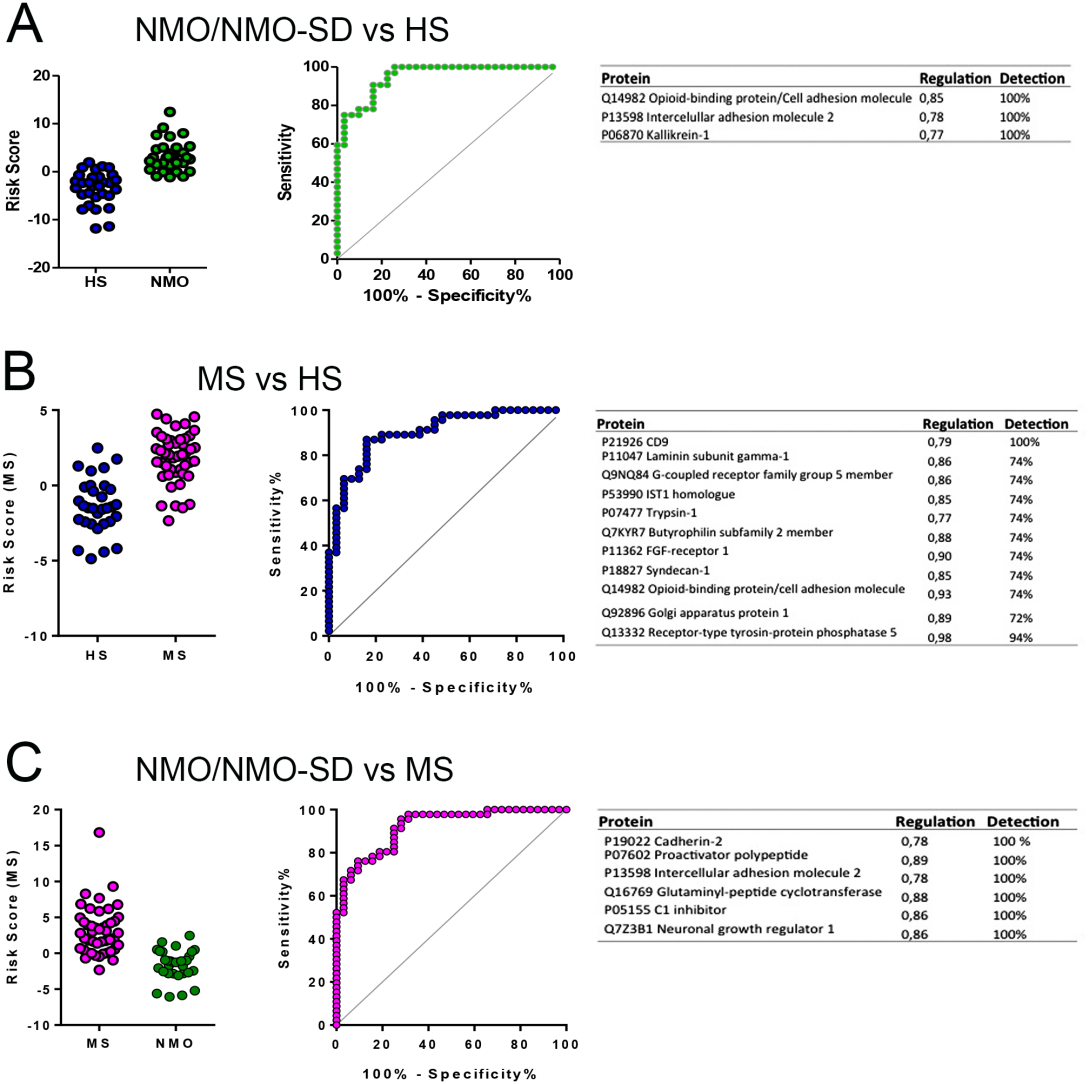
Risk scores by logistic regression. Risk scores and ROC curves for the discriminating profiles are shown. (A) A 3- protein profile based on the 333 proteins detected in all 109 was the optimal model (ROC AUC = 0.93, *p*<0.0001) for NMO/NMO-SD versus HS discrimination. (B) An 11-protein profile based on either proteins present in at least 80% of the samples in each group (520 proteins), or proteins present in at least 2 samples in each group (1021 proteins) was optimal for MS versus HS. (C) For NMO/NMO-SD versus MS discrimination, the best model was a 4-protein profile based on proteins present in at least 80% of the samples in each group (520 proteins).

Based on the 333 proteins detected in all 109 samples the classification analysis showed that a 3-protein profile was the optimal model (ROC AUC = 0.93, *p*<0.0001) for discriminating HS from NMO/NMO-SD with the protein score = 27.726 + (-17.035 x Q14982) + (-6.170 x P13598) + (-7.915 x P06870) (Fig 5A). Using 520 and 1021 proteins the best model was a 4-protein profile (ROC AUC = 0.915) including the above proteins in addition to glycoprotein integral membrane protein 1 (Q9NU53).

For MS versus HS discrimination, the best model (ROC AUC = 0.867, p = 0.0002) was an 11-protein profile derived from the data set with 520 proteins with the following expression: Score = 14.1606 + (-3.1307 x P21926) + (-3.7673 x P11047) + (5.1299 x Q9NQ84) + (0.3357 x Q7KYR7) + (-3.8180 x P53990) + (-0.6982 x P07477) + (-4.3527 x P11362) + (-4.1606 x P18827) + (-1.5233 x Q14982) + (-2.4859 x Q92896) + (4.1159 x Q13332) (Fig 5B). These proteins were also part of poorer performing profiles from the 333 and 1021 protein data sets (not shown).

For NMO/NMO-SD versus MS discrimination, the best model (ROC AUC = 0.858, *p*<0.0001) was derived from the 333-protein data set and was based on 6 proteins in the following expression: Score = -27.699 + (9.453 x P19022) + (8.301 x P07602) + (1.276 x P13598) + (1.202 x Q16769) + (6.582 x P05155) + (4.440 x Q7Z3B1) (Fig 5C). The two other data sets gave poorer performing models consisting of more proteins (not shown).

Thus, specific urine protein patterns could distinguish NMO/NMO-SD from MS and HS with a high discriminatory power (AUC >0.85 and *p*<0.0001 in all three cases).

## Discussion

In this study, we demonstrate that the urine proteome profile is different in patients with AQP4-seropositive NMO/NMO-SD compared to MS and HS. This is to our knowledge the first study to use high accuracy mass spectrometry to characterize the urine proteome of patients with NMO/NMO-SD and MS, although urine has been used as a source of biomarkers in other neurological diseases[13, 20].

Although new classification criteria of NMO/NMO-SD have been published very recently, which uses a unifying terminology of NMO-SD with or without AQP4 antibodies[21], in this paper we still use the widely accepted NMO/NMO-SD definition since patients were diagnosed based on the 2006 criteria[3].

Due to the high accuracy of the mass spectroscopy method used, we were able to identify a high number of proteins in the urine; one-fourth of these were shared by all samples. Quantitative analysis with univariate tests and statistical correction for multiple comparisons revealed that the NMO/NMO-SD proteome is useful for classification. Thus, we determined a total of 31 proteins, which were significantly different between NMO/NMO-SD and HS, 4 proteins were different between NMO/NMO-SD and MS, and 1 protein between MS and HS. In addition to the univariate analyses, the optimum combination of variables for discriminatory risk scores was determined by multivariate logistic regression. Out of the 31 proteins significantly different between NMO/NMO-SD and HS, a 3-protein combination defined a highly significant model for discriminating HS from NMO/NMO-SD. Likewise, the protein that could discriminate MS from HS was also part of the discriminating protein profile along with 10 other proteins. However, out of the 4 proteins that were significantly different between NMO/NMO-SD and MS, only 3 (cadherin–2, proactivator polypeptide and ICAM–2) turned out to be included in the 6-protein profile revealed by multivariate regression analysis.

The pathogenesis of the two diseases probably overlaps. Both are considered inflammatory demyelinating diseases although the primary immunopathogenesis may be different. In MS it is widely accepted that the inflammatory process is caused or propagated by an autoimmune cascade involving myelin reactive T cells, resulting in widespread demyelination and neuronal and axonal degeneration[22–24]. Deposition of pathogenic antibodies and complement activation may be more prominent in NMO, while axonal degeneration is less pronounced[1–3, 25]. However, MS is not a homogenous disease[24]. This can be one reason for the more heterogeneous proteome of the urine compared to both HS and NMO/NMO-SD. Indeed, an 11-protein profile was optimal for MS versus HS discrimination compared to the 3-protein and 6-protein profiles of NMO/NMO-SD versus HS and NMO/NMO-SD versus MS discrimination, respectively. Several distinct immunopathological profiles of MS have been suggested, even including humoral immune mechanisms to a variable degree[24, 26, 27]. B cells, plasma cells, autoantibodies and complement have been detected in MS lesions and in CSF[28, 29].

Among the proteins significantly differentiating NMO/NMO-SD from HS, 3 types of Ig chains were found to be upregulated (IgG_3_, Ig-κ, Ig-λ light chains). Ig consists of two identical heavy chains of α, γ, δ, ε or μ, and two identical light chains of κ or λ kappa or type [30]. The fact that we found the Ig-G3 heavy chain and the two light chains to be significantly increased in the urine of NMO/NMO-SD patients suggests an increased excretion of Igs or Ig fragments. However, rate nephelometry could confirm this increased excretion of Ig light chains, only in 3 out of 10 NMO/NMO-SD samples from cohort 1—all with high Ig content by LC-MS/MS, and in 1 of the 10 samples of an independent cohort. This lower sensitivity may be due to the lower sensitivity of immunonephelometric assays.

The fact that both heavy and light Ig chains were significantly increased in the mass spectrometry analysis of NMO/NMO-SD urines could indicate that specific antibodies or antibody fragments are increased. Considering that all NMO/NMO-SD patients in the present study were AQP4-seropositive, it is tempting to speculate that the specifically increased Igs, including the IgG_3_ are in fact AQP4-antibodies but this could not be confirmed by immunoassays. However, it is unlikely that intact antibody molecules are present in the urine samples, and the individual identifications of heavy and light chains may presumably originate from *in vivo*-fragmented immunoglobulin molecules. Since serum levels of anti-AQP4 antibodies have been shown to be elevated during relapse[31, 32], it is tempting to investigate if this is also reflected in the urine. However, as all patients in this study are stable on medication this falls beyond the scope of the study and would greatly limit the sample size of this group.

Many of the proteins that discriminated NMO/NMO-SD, MS and HS have been implicated in biological processes like leucocyte trafficking, blood-brain barrier breakdown, demyelination, myelin degradation and neuroinflammation. However, the majority of these were downregulated in the urine of patients with NMO/NMO-SD. Due to the highly sensitive technique we were still able to detect these downregulated proteins, but they are less likely to serve as potential biomarkers. Such downregulation also raises the question of increased protein degradation in the urine and/or sera of NMO/NMO-SD and MS patients. Extensive protein degradation, however, would have been expected to lead to fewer detected proteins in NMO/NMO-SD and MS samples, and this was not observed. In our proteomic setup the normalization of the samples was based on the protein concentration. Therefore, it is unlikely that variances in the quantitative protein content were responsible for such differences between NMO/NMO-SD, MS and HS. Furthermore, we excluded more than one third of the samples that were visibly contaminated by blood, or had high albumin/creatinine ratio or nitrite content to avoid patients with possible infection or kidney dysfunction. Nevertheless, considering that AQP4 is also expressed in the kidney and Ig chains were increased in the urine, it is tempting to speculate whether such decrease in particular proteins or altered degradation within the protein repertoire of the urine is indicative of an underlying subtle kidney dysfunction caused by AQP4 antibodies, which can be detected only by a very sensitive assay, such as LC-MS/MS. Indeed, cases with elevated creatinine kinase in the peripheral blood related to the clinical activity of NMO/NMO-SD have been reported, which may indicate a pathological role of AQP4-antibodies in extra-neuronal tissues expressing AQP4[33, 34].

Altogether, we identified around 1100 different proteins in the urine and one-third of these were found in all samples. These numbers largely agree with a previous work, where 1543 proteins including a large proportion of membrane proteins were identified in the urine of healthy donors[35]. In that study, protein identification was based on single peptide identifications (in addition to tandem mass spectra), while all protein identifications in the present study were more stringently based on the presence of at least 2 unique peptides. The former study identified 32 Ig species (and a number of hypothetical proteins of which some are of Ig nature) including the same distribution of κ and λ light chains that we observe in the present study, but none of the 3 specific significantly increased entries that we observe in the NMO/NMO-SD samples.

This is not study without limitations. First, using spot urine may have confounded the data analyses. Ideally this study would have been performed on fasting morning urine to eliminate any confounders due to changes in hydration or diet. By standardizing the protein content in the samples and discarding samples with low protein content we attempted to correct for differences in hydration. While we cannot adjust for any dietary differences, it is unlikely that great dietary variations exist between the groups. Furthermore any individual differences may only have served to undermine any statistical significant differences presented in this study

Second, the data interpretation may be confounded by the heterogenous medical treatments of the patient cohorts. The majority of samples from MS patients were collected just prior to the next treatment of natalizumab, meaning that the last injection would have been given 30 days before and is therefore less likely to be found in the urine as opposed to the therapies like interferon-beta with more frequent administrations.

Nevertheless, in a subanalysis, MS patients treated with natalizumab and interferon-beta were compared and we could not find significant differences in the urine proteome (not shown).

Third, only the Ig content of the urine has been validated in an independent cohort using a different method beside proteomics. Diagnostic classifiers should be validated in a large independent cohort consisting of both seronegative and seropositve patients including patients with active relapses. Furthermore specific proteins should be quantitated by immunoassays or single reaction monitoring mass spectrometric assays.

Despite these reservations, we here show that urine is an attractive source of biomarkers, which have so far been neglected in neurological contexts, despite its clear advantages over other body fluids. This study demonstrates that the use of high accuracy mass spectrometry in combination with multivariate regression analysis can reveal differences in the urine proteome that reflect differences in the pathology of distinct demyelinating diseases, and may serve as source of potential biomarkers.

## Supporting Information

S1 Table Proteins quantitied using 8-plex iTRAQ isobaric mass tags. Proteins extracted from urine samples collected from 32 NMO patients, 46 MS patients, and 32 healthy subjects (HS) were processed as described in the methods sections, randomly labelled with 8-plex iTRAQ isobaric tags (mass tags 116–119, 121)) and analyzed by nano-LC MS/MS. The individual samples were compared with a pool of the 32 HS samples (mass tag 113). The resulting ratios for each protein were used for students t-test calculation. Columns indicate Uniprot accession number, protein function, the percentage of matching amino acids from identified peptides, the sum of unique peptides used for identification, the average Mascot and Sequest protein score, the theoretical protein molecular weight, the calculated isoelectric point, t-test results, the total number of individuals included in the study including, the specific number of MNO, MS, and MS individuals, and the protein expression data for the identified proteins in the patient samples and healthy samples. Blanks indicate that the specific protein was not identified in the particular proteomic experiment.
(XLSX)

S2 Table Proteins identified by FDA to discriminate between NMO, MS and HS. The mean protein content of HS samples was normalized to 1. Table shows the regulation as a ratio of the mean of NMO and MS compared to HS as well as the percentage of samples the proteins was detected. *NMO*, *neuromyelitis optica; MS*, *multiple sclerosis; SPMS*, *secondary progressive MS; HS*, *healthy subjects*. *ND*, *not detected; SLAM*, *signal lymphocyte activation molecule; IL*, *interleukin; CSF; cerebrospinal fluid; MMP*, *matrix metalloprotease; TNFα*, *tumor necrosis factor alpha; experimental autoimmune encephalomyelitis*.
(DOCX)
