# Supplementary material for: The Urine Proteome Profile Is Different in Neuromyelitis Optica Compared to Multiple Sclerosis: A Clinical Proteome Study
Source: PLoS One. 2015 Oct 13;10(10):e0139659. doi: 10.1371/journal.pone.0139659 (PMC4604198; doi:10.1371/journal.pone.0139659)
Supplement: S2 Table — The mean protein content of HS samples was normalized to 1. Table shows the regulation as a ratio of the mean of NMO and MS compared to HS as well as the percentage of samples the proteins was detected. NMO, neuromyelitis optica; MS, multiple sclerosis; SPMS, secondary progressive MS; HS, healthy subjects. ND, not detected; SLAM, signal lymphocyte activation molecule; IL, interleukin; CSF; cerebrospinal fluid; MMP, matrix metalloprotease; TNFα, tumor necrosis factor alpha; experimental autoimmune encephalomyelitis. (DOCX) [file pone.0139659.s002.docx]

**Supplementary Table 1**

| Protein | NMO | | MS | | Biological significanse |
| --- | --- | --- | --- | --- | --- |
|  | Regulation vs HS | Detection  rate | Regulation vs HS | Detection  rate |  |
| **Proteins discriminating NMO from HS** |  |  |  |  |  |
| O75882 Attractin | 0.92 | 100% | 0.97 | 100% | Type C lectin is expressed on human peripheral blood monocytes and may influence monocyte function. |
| O00462 Beta-mannosidase | 0.86 | 100% | 0.97 | 74% | Localized in the lysozome, involved in mannose catabolism. Decreased activity in the CSF of MS patients. |
| P19835 Bile salt-activated lipase | 0.77 | 100% | 0.93 | 100% | Found in pancreas juice and breast milk. |
| P19022 Cadherin-2 | 0.78 | 100% | 0.96 | 100% | Neural cadherin, transmembrane protein involved in cell adhesion.  Expressed in astrocytes at the blood-brain-barrier and in sprouting neurons.  Seen in early remyelination in experimental autoimmune encephalomyelitis (EAE). |
| Q9H159 Cadherin-19 | 0.83 | 92% | 0.92 | 66% | Expressed in myelin-forming cells in chicken and rat. Possible marker for gliomas. |
| P21926 CD9 | 0.82 | 100% | 0.79 | 100% | Surface protein in lymphohematopeotic cells and myelin. Reduced in demyelinated lesions and increased on blood vessels in lesion areas. |
| Q9NZZ3 Charged multivesicular body protein 5 | 0.86 | 100% | 0.93 | 100% | Member of the multivesicular body, which serves as an anti-apoptotic protein. |
| P39060 Collagen alpha-1(XVIII) chain | 0.87 | 100% | 0.90 | 100% | Extracellular matrix protein, may play an important role in retinal structure and in neural tube closure. |
| Q12860 Contactin-1 | 0.91 | 100% | 0.96 | 100% | Neuronal membrane protein that functions as cell adhesion molecule, increased in the CSF of MS patients. |
| P11362 Fibroblast growth factor receptor 1 | 0.83 | 100% | 0.90 | 74% | Receptor tyrosine kinase whose ligands are specific members of the fibroblast growth factor family.  Expressed in the cuprizone and EAE models.  Expressed in oligodendrocyte precursor cells in MS lesions.  Increased in CSF of MS patients compared to NMO. |
| Q12841 Follistatin-related protein 1 | 0.80 | 97% | 0.91 | 72% | Proinflammatory molecule, secreted by neurons and endothelial cells and mesenchymal derived cells by IL-1β, TNFα, and IL-6. Enhances the ability of T cells and monocytes/macrophages to respond to inflammatory signals. |
| Q9ULV1 Frizzled-4 | 0.83 | 89% | 0.97 | 66% | Regulates vascular development of retina and the retina-blood barrier. |
| Q16769 Glutaminyl-peptide cyclotransferase | 0.88 | 100% | 0.99 | 100% | Expressed in neurons in normal human and murine brains and in brains of patients with Alzheimer’s disease.  Stabilize peptides from degradation by aminopeptidases, normal part of CSF in HS and MS patients. |
| Q9NU53 Glycoprotein integral membrane protein | 0.80 | 89% | 0.90 | 70% | Integral membrane protein |
| P01860 Ig gamma-3 chain C region | 1.26 | 92% | 1.01 | 70% | Immunoglobulin |
| P04433 Ig kappa chain V-III region | 1.09 | 100% | 1.12 | 100% | Immunoglobulin |
| P0CG05 Ig lambda-2 chain C regions | 1.18 | 100% | 1.13 | 100% | Immunoglobulin |
| Q08334 IL-10 receptor subunit beta | 0.82 | 87% | 0.96 | 72% | Accessory chain essential for the active IL-10 receptor, potent anti-inflammatory cytokine in MS. Found in elevated levels in serum of NMO patients compared to MS. |
| Q16270 Insulin-like growth factor-binding protein 7 | 0.85 | 100% | 0.92 | 100% | Regulates the availability of insulin-like growth factors and modulates receptor binding. Involved in memory consolidation and possible biomarker in Alzheimer’s disease. |
| P35858 Insulin-like growth factor-binding protein complex acid labile subunit | 0.83 | 97% | 0.96 | 96% | Maintains the integrity of the circulating insulin-like growth factor system. Deficiency associated with mild growth retardation. |
| P13598 Intercellular adhesion molecule 2 | 0.78 | ND | 0.94 | 74% | Cell adhesion molecule, lymphocyte trafficking  Expressed in blood vessels of MS lesions. |
| Q86YT9 Junctional adhesion molecule-like | 0.82 | 97% | 1.02 | 94% | Costimulatory receptor for epithelial γδ T cell activation. Expressed in monocytes, upregulated by monocyte chemotaxis protein-1 stimulation. Expressed on neurophil and regulates transepithelial migration. |
| P06870 Kallikrein-1 | 0.77 | 100% | 0.91 | 100% | Cleaves kininogen to bradykinin.  Expressed in endothelial cells and produces bradykinin that acts on neurons and astrocytes and as a chemoattractant of microglia in multiple sclerosis. Activates matrix metallopeptidase (MMP) 2 and MMP9.  Associated with neurodegeneration in SPMS. |
| P11047 Laminin subunit gamma-1 | 0.81 | 100% | 0.86 | 74% | Major noncollagenous constituent of the basal membrane. Implicated in blood brain barrier, cell adhesion, differentiation, migration, signaling and neurite outgrowth. |
| Q7Z3B1 Neuronal growth regulator 1 | 0.86 | 100% | 0.96 | 100% | Associated with obesity. |
| Q9P121 Neurotrimin | 0.85 | 97% | 0.98 | 70% | Part of the IgLON family of neural cell adhesion molecules together with opioid bindings protein. |
| Q14982 Opioid-binding protein/cell adhesion molecule | 0.85 | 100% | 0.93 | 74% | Accessory role in opioid receptor binding, part of the neurit growth family.  Found in gray matter in rat.  Part of the IgLON subfamily of neural cell adhesion molecules together with neurotrimin. |
| Q96JQ0 Protocadherin-16 | 0.91 | 100% | 0.98 | 100% | Expressed in neurites, dendrites and synapses. |
| Q13332 Receptor-type tyrosine-protein phosphatase S | 1.05 | 97% | 0.98 | 94% | Implicated in the molecular control of adult nerve repair and spinal cord injury. |
| Q9UIB8 SLAM family member 5/ Signaling lymphocytic activation molecule 5 | 0,87 | 100% | 0,99 | 74% | Expressed on B lymphocytes, monocytes, T cells and platelets.  Increases proliferative responses of activated T-cells. Enhances interferon gamma secretion in lymphocytes and induces platelet stimulation. |
| F5H265 Ubiquitin | 0.79 | 76% | 0.91 | 54% | Regulatory protein found in all cells, downregulated in the CSF of MS patients. |
| **Proteins discriminating NMO from MS** |  |  |  |  |  |
| P19022 Cadherin-2 | 0.78 | 100 % | 0.92 | 100% | Neural cadherin, transmembrane protein involved in cell adhesion.  Expressed in astrocytes at the blood brain barrier.  Expressed in sprouting neurons.  Seen in early remyelination during EAE. |
| P01860 Ig-Gamma3-chain C region | 1.26 | 92% | 1.01 | 89% | Immunoglobulin |
| P13598 Intercellular adhesion molecule 2 | 0.78 | 100% | 0.94 | 74% | Cell adhesion molecule involved in lymphocyte trafficking.  Expressed in blood vessels of MS lesions. |
| P07602 Proactivator polypeptide/prosaposin | 0.89 | 100% | 1.02 | 100% | Participate in the lysosomal degradation of sphingolipids, which takes place by the sequential action of specific hydrolases. e.g. gangliosides in the CNS.  Myelinotrophic and neurotrophic factor. |
| **Proteins discriminating NMO from MS** |  |  |  |  |  |
| P21926 CD9 | 0.82 | 100% | 0.79 | 100% | Surface protein in lymphohematopeotic cells and myelin. Reduced in demyelinated lesions and increased on blood vessels in lesion areas. |

The mean protein content of HS samples was normalized to 1. Table shows the regulation as a ratio of the mean of NMO and MS compared to HS as well as the percentage of samples the proteins was detected. *NMO, neuromyelitis optica; MS, multiple sclerosis; SPMS, secondary progressive MS; HS, healthy subjects. ND, not detected; SLAM, signal lymphocyte activation molecule; IL, interleukin; CSF; cerebrospinal fluid; MMP, matrix metalloprotease; TNFα, tumor necrosis factor alpha; experimental autoimmune encephalomyelitis.*
